# Characterization of Effective Half-Life for Instant Single-Time-Point Dosimetry Using Machine Learning

**DOI:** 10.2967/jnumed.124.268175

**Published:** 2025-05

**Authors:** Carlos Vinícius Gomes, Yizhou Chen, Isabel Rauscher, Song Xue, Andrei Gafita, Jiaxi Hu, Robert Seifert, Lorenzo Mercolli, Julia Brosch-Lenz, Jimin Hong, Marc Ryhiner, Sibylle Ziegler, Ali Afshar-Oromieh, Axel Rominger, Matthias Eiber, Thiago Viana Miranda Lima, Kuangyu Shi

**Affiliations:** 1 Department of Nuclear Medicine, Inselspital, Bern University Hospital, University of Bern, Bern, Switzerland;; 2 Institute of Radiology and Nuclear Medicine, Lucerne University Teaching and Research Hospital, Lucerne, Switzerland;; 3 Graduate School for Cellular and Biomedical Sciences, University of Bern, Bern, Switzerland;; 4 Department of Nuclear Medicine, School of Medicine, Technical University Munich, Klinikum Rechts der Isar, Munich, Germany;; 5 Division of Nuclear Medicine, Department of Biomedical Imaging and Image-Guided Therapy, Vienna General Hospital, Medical University of Vienna, Vienna, Austria;; 6 Ahmanson Translational Theranostics Division, Department of Molecular and Medical Pharmacology, UCLA, Los Angeles, California;; 7 Institute of Nuclear Medicine, Glen Burnie, Maryland;; 8 Department of Nuclear Medicine, Hannover Medical School (MHH), Hannover, Germany;; 9 Department of Nuclear Medicine, LMU University Hospital, LMU Munich, Munich, Germany; and; 10 Faculty of Health Sciences and Medicine, University of Lucerne, Lucerne, Switzerland

**Keywords:** radiopharmaceutical therapy, [^177^Lu]Lu-PSMA I&T, machine learning, single time point, dosimetry

## Abstract

Single-time-point (STP) image-based dosimetry offers a more convenient approach for clinical practice in radiopharmaceutical therapy (RPT) compared with conventional multiple-time-point image-based dosimetry. Despite numerous advancements, current STP methods are limited by the need for strict and late timing in data acquisition, posing challenges in routine clinical settings. This study introduces a new concept of instant STP (iSTP) dosimetry, achieved by predicting the effective half-life (*T*_eff_) of organs using machine learning applied on pretherapy patient data (PET and clinical values). **Methods:** Data from 22 patients who underwent pretherapy ^68^Ga-gallium *N*,*N*-bis[2-hydroxy-5-(carboxyethyl)benzyl]ethylenediamine-*N*,*N*-diacetic acid ([^68^Ga]Ga-PSMA-11) imaging and subsequently [^177^Lu]Lu-PSMA I&T RPT were analyzed. A machine learning model was developed for *T*_eff_ predictions for the left and right kidneys, liver, and spleen subsequently used to estimate time-integrated activity and absorbed dose. iSTP results were compared against multiple-time-point and previously proposed Hänscheid methods. Our method comprised 2 different prediction scenarios, using data before each therapy cycle and from the first cycle. **Results:** The iSTP method introduced early posttherapy time points (2, 20, 43, and 69 h) for the left kidney, right kidney, liver, and spleen. Dosimetry in the first scenario, aggregating 2 and 20 h, achieved mean differences in time-integrated activity below 27% for all organs. To assess the feasibility, these time points were compared with the best results from the Hänscheid method (kidneys, 69 h; liver and spleen, 20 h). At 2 h, a significant difference (*P* < 0.001) was found for almost all organs except for the spleen (*P* = 0.1370). However, at 20 h, no significant differences were found for the right kidney, liver, and spleen, apart from the left kidney (*P* < 0.01). In the scenario using only the initial PET/CT data to predict *T*_eff_ for subsequent cycles, iSTP dosimetry achieved no statistical significance (*P* > 0.05) for all cycles in comparison to results using PET data before each therapy cycle. **Conclusion:** Our preliminary results prove the concept for prediction of *T*_eff_ with pretherapy data and achieving STP shortly and flexibly after the RPT. The proposed method may expedite the application of dosimetry in broader contexts, such as outpatient or short-duration inpatient treatment.

## Visual Abstract

**Figure:**
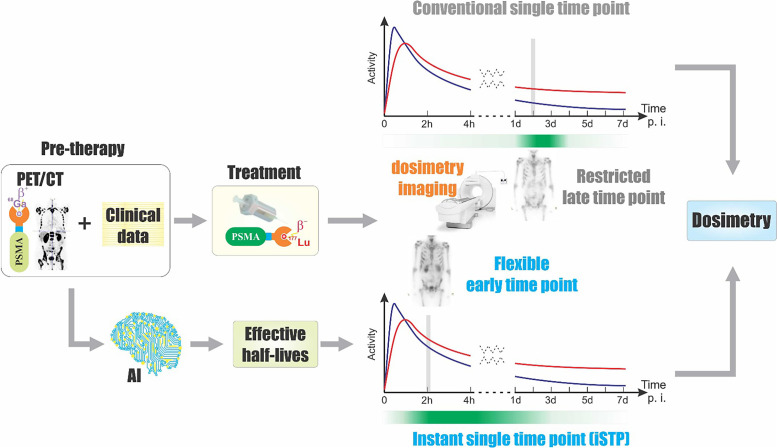

Dosimetry in radiopharmaceutical therapies (RPTs) is essential to ensure that treatments are effective and safe, which is based on extensive research and clinical trials (1–4). The accepted approach to account for the radiopharmaceutical biodistribution relies on the acquisition of multiple SPECT images at different time points after injection of the radiopharmaceutical, known as the multiple-time-point (MTP) method. Challenges of this method include limited resources, high costs, scarce scanner availability, and patient burdens, potentially increasing overall treatment costs (5–9). Despite these hurdles, evidence underscores dosimetry’s role in tailoring treatment strategies more precisely (10,11).

The adoption of single-time-point (STP) image-based dosimetry instead of the MTP method has become accepted and pursued alternatively because of its practicality based on 1 single posttherapy scan. The STP methods proposed by Madsen et al. (5) and Hänscheid et al. (12), which initially focused on neuroendocrine tumors, were expanded to prostate-specific membrane antigen (PSMA)-targeting radiopharmaceuticals (8,9). However, this method presents time-dependency limitation that would influence its clinical use (5–8,12–15). Hou et al. cautioned about the method’s accuracy and emphasized its dependence on both radiopharmaceutical and patient specifics (14). Additionally, Brosch-Lenz et al. (8) and Hou et al. (14) suggested that STP dosimetry might not suit all organs or tissues in specific therapeutic contexts. Furthermore, the decision of whether therapy-personalized dosimetry should focus on sparing organs at risk or maximizing lesion absorbed dose remains a point of consideration. This is particularly relevant in the context of significant variations in tumor effective half-lives (*T*_eff_), for which the limitations of STP approaches might be more pronounced, as previously documented (14).

Current limitations in STP dosimetry, particularly in commercial applications, stemmed largely from their dependence on the time point of the SPECT scan for dose evaluation. Kratochwil et al. (16) suggested that scintigraphy performed 1–2 d after infusion served as an effective imaging follow-up for PSMA-positive lesions, reinforcing the relevance of a single scan for dosimetry. Additionally, differences in patient discharge might influence the availability of late-imaging time points. Patients stay in the hospital for a 48-h period in Switzerland during which imaging could be performed. On the other hand, patients receive posttherapeutic scans 24 h after each treatment cycle in Singapore (17).

To address these challenges, the present article introduces an instant STP (iSTP) method based on pretherapy patient data, building on the assumption that the *T*_eff_ is a patient-intrinsic characteristic (8,14,18), allowing flexible dosimetry with a single measurement during an early scan time point spanning from day 0 to day 3 after injection of the therapy. Our development aims to achieve flexible and iSTP dosimetry after RPT.

## MATERIALS AND METHODS

### Patient Cohort and Dosimetry Methods

#### Patient Samples

Twenty-two patients with metastatic castration-resistant prostate cancer underwent [^177^Lu]Lu-PSMA I&T (^177^Lu-PSMA I&T; mean injected activity, 7.3 ± 0.3 GBq) RPT at Klinikum Rechts der Isar, Technische Universität München, Germany. In total, 44 therapy cycles were included, comprising pretherapy ^68^Ga-gallium *N*,*N*-bis[2-hydroxy-5-(carboxyethyl)benzyl]ethylenediamine-*N*,*N*-diacetic acid (^68^Ga-PSMA-11; mean injected activity, 119.0 ± 25.1 MBq) PET/CT scans before each therapy cycle (1, 2, 3, 4, 5, and 9) and SPECT/CT scans at MTPs after injection, further referred to as *t*_sc_ in hours (at 2, 20, 43, 69, 144, and >165 h, with a minimum of 3 and a maximum of 5 time points). This dataset has been previously reported (19,20). The general characteristics of the patients are detailed in Table 1. In accordance with the local ethics committees in Germany, the institutional review board of the Technische Universität München (reference no. 115/18) approved this study, and all subjects provided written informed consent. All patient data were pseudoanonymized.

**TABLE 1.** General Characteristics of Patients per Therapy Cycle, Represented as Mean Values of Age, Weight, PSA Level, LDH, Creatinine, and Hemoglobin

	Characteristics per cycle			
Parameter	Cycle 1	Cycle 2	Cycle 3	Cycle ≥4
Number of patients	20	12	6	4
Age (y)	69 ± 9	69 ± 9	69 ± 9	69 ± 9
Weight (kg)	82.3 ± 10.6	82.3 ± 10.6	82.3 ± 10.6	82.3 ± 10.6
PSA (ng/mL)*	714.59	591.35	611.67	438.19
LDH^†^	328.05	275.25	270.83	247.67
Creatinine^‡^	1.0	0.9	0.9	0.9
Hemoglobin^§^	11.52	10.78	10.45	10.72

* PSA levels minimum and maximum values, 0.1 and 2936 ng/mL.

† LDH minimum and maximum values, 161.0 and 664.0.

‡ Creatinine minimum and maximum values, 0.5 and 2.0.

§ Hemoglobin minimum and maximum values, 8.7 and 15.2.

PSA = prostate-specific antigen; LDH = lactate dehydrogenase.

Supplemental Table 1 details the number of patients grouped by cycle and time points (supplemental materials are available at http://jnm.snmjournals.org).

#### Data Acquisition and Preparation

Volumes of interest including the left kidney, right kidney, liver, spleen, and urinary bladder were segmented from pretherapy CT images, from which the SUV_mean_ of the organs was extracted. From posttherapy CT images, the left kidney, right kidney, liver, and spleen were used for dosimetric analysis. Segmentation and quantification were performed using SurePlan MRT (MIM Software), requiring manual corrections when necessary (www.mimsoftware.com).

#### Time-Integrated Activity (TIA): MTP

The pharmacokinetics of ^177^Lu-PSMA I&T were modeled separately in the left and right kidneys, liver, and spleen, using the following biexponential function:

$$A\left(t\right)=A_{1}e^{-(\lambda_{1}+\lambda_{\mathrm{phys}})t}-{A_{2}e}^{-\left(\lambda_{2}+\lambda_{\mathrm{phys}}\right)t},$$ Eq. 1

where *A*_1_ is the organ activity (MBq), *A*_2_ is the initial activity, λ_1_ and λ_2_ are the biological decay constants, and λ_phys_ is the physical decay of ^177^Lu (6.65 d). Parameters *A*_2_ and λ_2_ were considered as population mean data, as described in Supplemental Table 2. Equation 1 was fitted to cycles with at least 3 posttherapeutic SPECT time points to estimate the *T*_eff_ and TIA (also referred as $\overset{∼}{A}$) (Eq. 2):

$$\overset{∼}{A}=\int_{0}^{\infty }A_{1}e^{-(\ln\;\left(2\right)/T_{\mathrm{eff}})t}-{A_{2}e}^{-\left(\lambda_{2}+\lambda_{\mathrm{phys}}\right)t}dt.$$ Eq. 2

### TIA: STP

Two approaches have been selected to estimate TIA: characterization by the predicted *T*_eff_ and an initial activity (*A*_0_) (Eq. 3) and utilization of the method by Hänscheid et al. (Eq. 4) (12). Note that time point *t*_sc_ refers to the specific time point of the SPECT image acquisition in hours. A detailed demonstration of ${\overset{∼}{A}}_{\mathrm{iSTP}}$ can be found in the supplemental materials.

$${\overset{∼}{A}}_{\mathrm{iSTP}}=\int_{0}^{\infty }A_{0}{\times e}^{-(\ln\;\left(2\right)/T_{\mathrm{eff}})t}dt,$$ Eq. 3

$${\overset{∼}{A}}_{H}=\;\frac{1}{\ln\;2}\times A\left(t_{\mathrm{sc}}\right)\times 2\times t_{sc}.$$ Eq. 4

**FIGURE 1.**
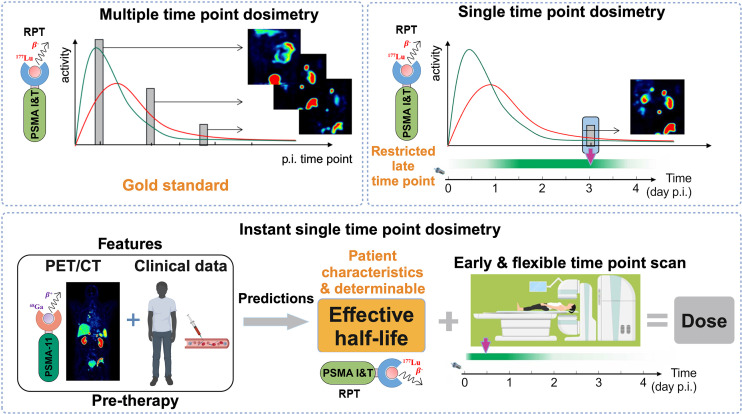
Concept of iSTP dosimetry in radiopharmaceutical therapy, encompassing MTP and STP methods. p.i. = postinjection.

The STP methods were henceforth referred to as iSTP and Hänscheid, as illustrated in Figure 1. However, the Hänscheid method was developed to incorporate scan time points within a range of 0.75 to 2.5 times the *T*_eff_ of an organ (including 20, 43, and 69 h *t*_sc_). It is important to note that the calculated organ values at 2 h *t*_sc_ for this method were used in this study for reference only.

#### Absorbed Dose

Dosimetry was performed for the left kidney, right kidney, liver, and spleen using the iSTP and Hänscheid methods (www.opendose.org). The absorbed dose ($D\left(r_{T},\;T_{D}\right)$), in Grays, in a given target region ($r_{T}$) over a dose-integration period ($T_{D}$), referred by the product between the $\overset{∼}{A}\left(r_{S},\;T_{D}\right)$, in the source tissue ($r_{S}$), and the absorbed dose per decay ($S(r_{T}\leftarrow r_{S})$) (21); is determined:

$$D\left(r_{T},\;T_{D}\right)=\;\sum_{r_{S}}\overset{∼}{A}\left(r_{S},\;T_{D}\right)S(r_{T}\leftarrow r_{S}).$$ Eq. 5

### Machine Learning–Based Model: Development and Validation

The machine learning–based model was developed by incorporating PET imaging quantification, clinical features, and the correspondent *T*_eff_ of the target organ (left and right kidneys, liver, and spleen). For each specific organ (left and right kidneys, liver, and spleen), a data matrix was defined on the basis of the patient cycle. In this matrix, an individual patient cycle is represented by rows, and their corresponding features form the columns. The selected features (*F_i_*) for the organs (supplemental materials) were derived from not only basic patient characteristics but also pretherapy PET/CT metrics, such as organ SUV_mean_. In addition, the matrix integrated relevant clinical data (Table 1), all of which are curated and structured according to organ ${T_{\mathrm{eff}}}_{i}$ function:

$$T_{{\mathrm{eff}}_{i}}=\mathrm{SVR}\left({F_{i}}_{\left\{\mathrm{patient}\;\mathrm{characteristic},\;\;\mathrm{PET}\;\mathrm{data},\;\;\mathrm{clinical}\;\mathrm{data}\right\}}\right).$$ Eq. 6

We used support vector regression (SVR) (Scikit-learn) as a predictive method for regression, using a radial basis function kernel to construct a hyperplane in high-dimensional space while minimizing error and controlling model complexity. To enhance generalization, standard scaling was applied to the features, ensuring zero mean and unit variance. The hyperparameters *C* and ϵ were optimized to balance prediction accuracy and model robustness, preventing overfitting and effectively capturing underlying data trends. The SVR was chosen because it provided a superior fit for the small datasets involved in this study (22,23).

The model validation was conducted using k-fold cross-validation. The dataset was partitioned into a *k* of 10 folds. The error function for model performance evaluation was computed as the mean percentage error between the predicted and measured *T*_eff_. Ultimately, the performance was summarized by calculating the mean and SD of the mean error (ME) across the folds.

### Clinical Robustness

The robustness of the developed iSTP model was evaluated in 2 scenarios: a model dependency on the time points of the posttherapy SPECT scan, which is a known limitation of the currently used STP method, and a model dependency on the pretherapy ^68^Ga-PSMA-11 PET/CT before the therapy cycle.

The first scenario computed the organ TIA dependence on the time point of the ^177^Lu-PSMA I&T SPECT/CT. Our method included pretherapeutic data before each cycle as a feature, as indicated in Equation 6.

The second scenario of our study evaluated the case in which PET/CT pretherapy imaging would be performed only once for the entire treatment. For this purpose, we used pretherapy data from the first cycle to train our machine learning model for predicting subsequent cycles.

### Analysis

To estimate the relative absolute differences (RADs), we used the following equation:

$$\mathrm{RAD}=\left|\frac{\theta_{\mathrm{STP}}}{\theta_{\mathrm{MTP}}}-1\right|\times 100\%,$$ Eq. 7

where θ_MTP_ and θ_STP_ are estimated values in TIA and absorbed dose from the standard MTP and STP (iSTP and Hänscheid) methods, respectively. Statistical significance was determined at a *P* value of less than 0.05; otherwise, the results were considered nonsignificant.

## RESULTS

### Dosimetry and *T*_eff_

The MTP-based absorbed doses and *T*_eff_ results that served as the reference gold standard are shown in Table 2, used for the evaluation of different STP methods.

**TABLE 2.** Absorbed Dose and *T*_eff_ Results for Left Kidney, Right Kidney, Liver, and Spleen, Presented as Mean *±* SD, with Minimum and Maximum Values

Organ	Absorbed dose (Gy)	*T*_eff_ (h)
Left kidney	3.7 *±* 1.9 (0.8–8.3)	35 *±* 16 (7–65)
Right kidney	3.7 *±* 1.9 (0.9–8.6)	34 *±* 16 (7–65)
Liver	0.07 *±* 0.04 (0.02–0.23)	11 *±* 4 (6–25)
Spleen	0.7 *±* 0.4 (0.2–1.6)	9 *±* 4 (5–25)

### iSTP: Machine Learning–Based Model Development and Validation

**FIGURE 2.**
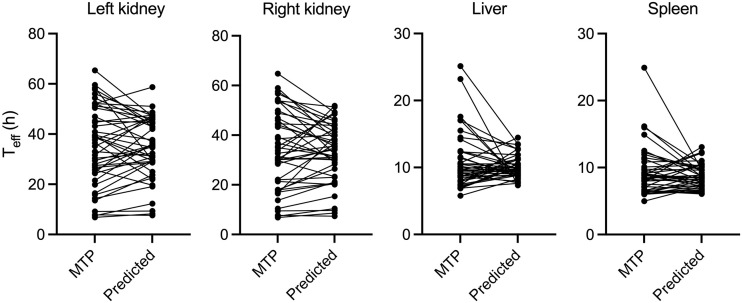
Relation between MTP and predicted *T*_eff_ for left kidney, right kidney, liver, and spleen across all therapy cycles.

Based on our model’s predictions, the SVR model estimated the organ’s *T*_eff_ with an ME of 24.4% ± 7.4% for the left kidney, 27.2% ± 10.0% for the right kidney, 22.7% ± 9.8% for the liver, and 19.7% ± 5.4% for the spleen. Figure 2 shows the relation of *T*_eff_ results, depicting the MTP and the predicted values. Statistical analysis using the Wilcoxon signed-rank test indicated no statistical significance for all organs (*P* = 0.55, 0.28, 0.87, and 0.89 for the left kidney, right kidney, liver, and spleen, respectively).

### STP: Time-Point Independency

Dosimetry was conducted (Eq. 5), wherein the respective TIAs were computed using 2 distinct methods: the iSTP model by applying the predicted *T*_eff_ (Eq. 3) and the Hänscheid method (Eq. 4). The results in the TIA, presented as RADs per organ, were benchmarked against MTPs (Eq. 7).

The iSTP method, at 2, 20, 43, and 69 h *t*_sc_, resulted in mean RADs in TIA of 19.5% ± 15.4% and 22.3% ± 17.4% for the left and right kidneys, respectively. Differences of 21.2% ± 18.9% and 23.4% ± 22.5% were observed for the liver and spleen, respectively. However, the Hänscheid method presented RADs of 18.2% ± 13.5% for the left kidney and 17.4% ± 13.9% for the right kidney, followed by 17.8% ± 16.4% and 27.4 ± 20.3% for the liver and spleen, respectively, considering 20, 43, and 69 h *t*_sc_. The nonpaired Mann–Whitney test was performed for all organs, showing statistically nonsignificant differences (*P* > 0.05).

### Robustness: Time-Point Dependency

**FIGURE 3.**
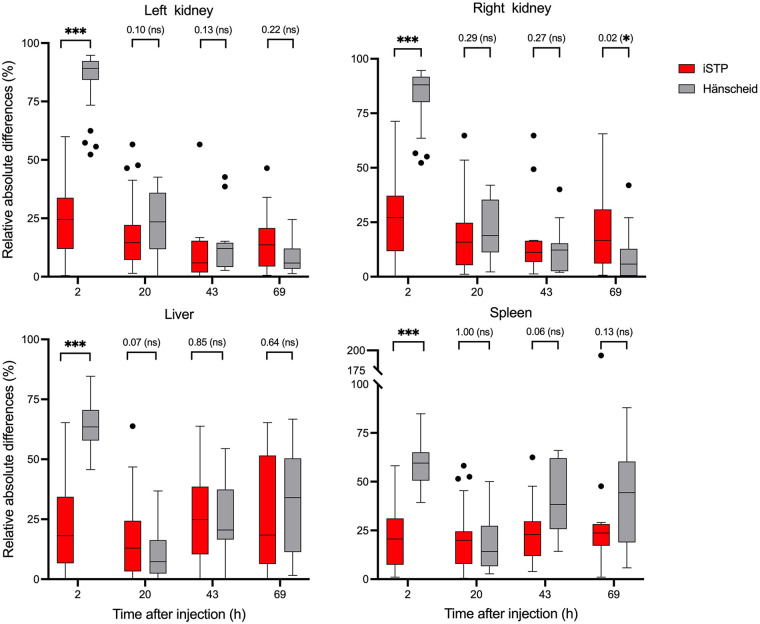
RADs in TIA by organ for both STP (iSTP and Hänscheid) methods compared with MTP method. Illustrated for left and right kidney, liver, and spleen at earlier postinjection time points (2 h*, 20 h, 43 h, and 69 h). *2 h *t*_sc_ is not applicable to Hänscheid method; included for comparison purposes. ***P < 0.001. ns = not significant.

RADs in TIA were calculated from the iSTP and Hänscheid methods against the MTP organized per early time point after injection (aggregating 2, 20, 43, and 69 h) for the left kidney, right kidney, liver, and spleen. Figure 3 shows the RAD values for both methods along with the statistical analysis.

Data for all organs were analyzed using the iSTP method. For the left kidney, at 2 h, this method showed a mean RAD of 25.0% ± 15.9%, which decreased to 17.4% ± 13.5% at 20 h. The lowest errors were observed at 43 h (11.0% ± 15.5%) and 69 h (15.0% ± 13.5%) *t*_sc_.

The iSTP performed similarly for both kidneys. For the right kidney, at 2 h *t*_sc_, a mean difference of 27.3% ± 17.0% was observed. At 20, 43, and 69 h, the RAD errors were 18.6% ± 15.6%, 17.3% ± 19.5%, and 20.6% ± 19.8%, respectively.

Regarding the liver, this method presented a RAD of 22.1% ± 19.3% at 2 h *t*_sc_. At 20 h *t*_sc_, a lower RAD of 16.3% ± 14.8% was observed, followed by RAD values of 27.1% ± 20.6% at 43 h and 27.2% ± 24.3% at 69 h. Results for the spleen showed a mean RAD, with values ranging from 20.2% ± 14.9% at 20 h, reaching the highest recorded value of 35.5% ± 48.8% at 69 h *t*_sc_.

The comparison between the iSTP and Hänscheid methods, including at 20, 43, and 69 h, showed no statistically significant differences across the assessed time points, except for the right kidney at 69 h *t*_sc_ (*P* = 0.02). For the left kidney at 20 h, the Hänscheid method achieved a mean RAD of 23.1% ± 13.1%, followed 13.8% ± 13.3% and 8.1% ± 6.3% at 43 and 69 h, respectively. A similar pattern was observed for the right kidney, with 9.3% ± 12.4% at 69 h *t*_sc_. In liver and spleen measurements, the Hänscheid method obtained optimal RADs of 10.6% ± 9.0% and 17.6% ± 11.8%, respectively, at 20 h *t*_sc_.

Furthermore, we compared the iSTP early time points (2 and 20 h) for all organs with the optimal Hänscheid *t*_sc_ achieved at 69 h for the left and right kidneys and at 20 h for the liver and spleen. These findings are detailed in the Discussion section and are depicted in Supplemental Figure 1.

### Robustness: Cycle Dependency

**FIGURE 4.**
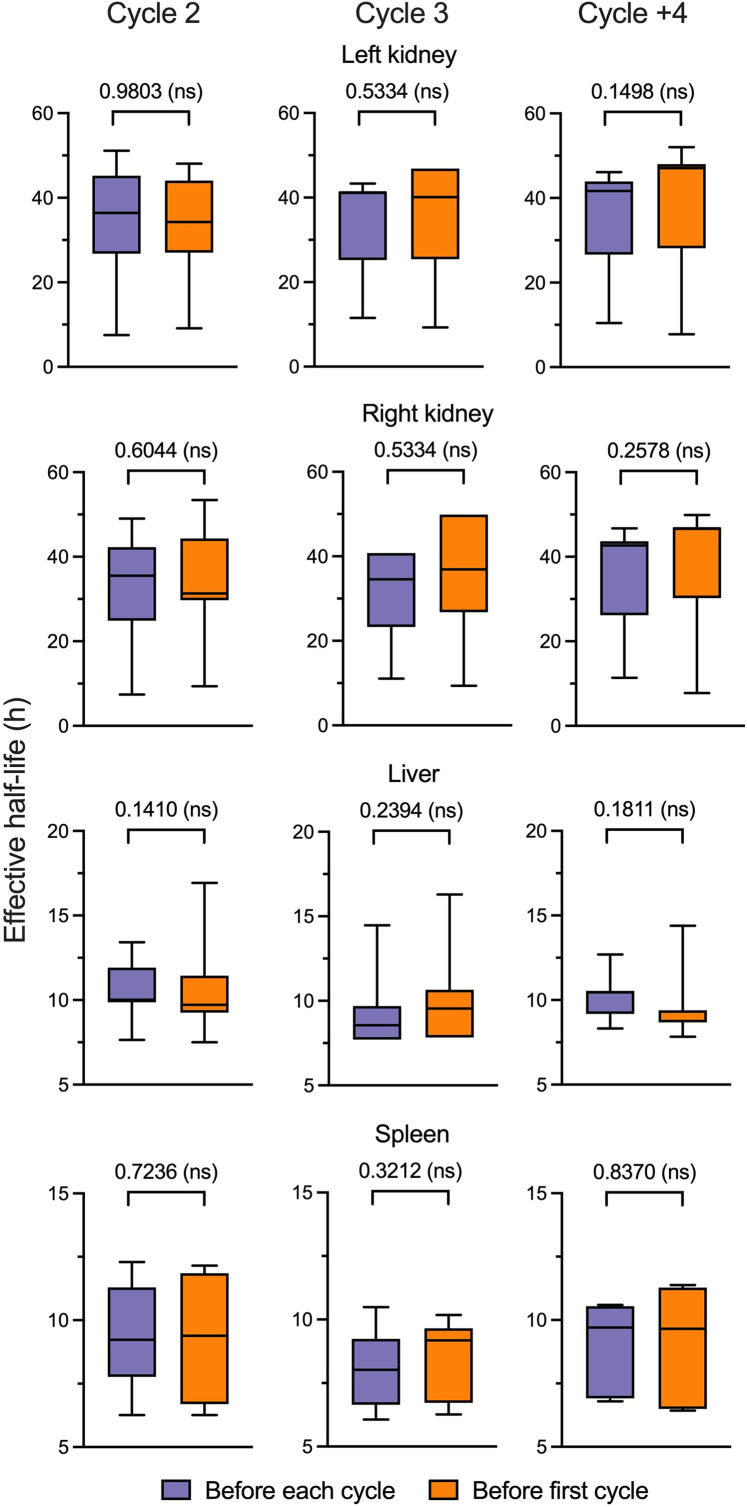
Statistical correlation between predicted *T*_eff_ when using pretherapy data before each cycle and before first cycle. Nonpaired Mann–Whitney test was performed for each therapy cycle, represented by columns, for left kidney, right kidney, liver, and spleen. ns = not significant.

In the second scenario, we introduced the first cycle’s ^68^Ga-PSMA-11 PET data to predict *T′*_eff_ for all subsequent therapy cycles, estimating the RAD in the absorbed dose. Two patient cycles were excluded because of missing information from the first cycle. The *T′*_eff_ SVR model aggregated ME predictions of 27.8% ± 10.0%, 32.5% ± 10.1%, 24.1% ± 11.1%, and 20.5% ± 7.2% for left and right kidneys, liver, and spleen, respectively. The nonpaired Mann–Whitney test, as shown in Figure 4, revealed no statistically significant differences (*P* > 0.05) in cycles 2, 3, and 4 or more across organs when comparing the predicted *T*_eff_ using pretherapy data obtained before each therapy cycle with the predictions from before the first-cycle scenario.

**FIGURE 5.**
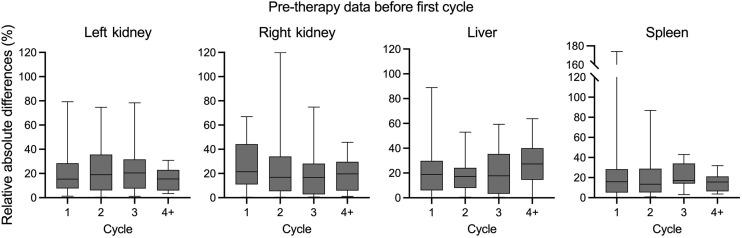
RADs in absorbed dose calculated and grouped by cycle using iSTP model from data preceding first therapy cycle.

The estimated RADs in absorbed dose are illustrated in Figure 5, obtained from the second scenario. The differences by organ, categorized according to cycle, spanned from 14.6 *±* 8.7% up to 30.8 *±* 19.8% across all organs. Organ results were compared between both scenarios, showing no significant differences, consistently yielding *P* values greater than 0.05 (Supplemental Fig. 2).

## DISCUSSION

A critical challenge in the practice of RPT is the dilemma of precision and clinic-friendly techniques. Finding a practical solution to implement dosimetry-guided personalization is difficult while allowing wide adoption in diverse routine scenarios. Particularly, outpatient treatment has become a mainstream practice in many countries, for which the conventional MTP method is unable to provide a flexible and convenient solution because of the strict requirement for a time point imaging acquisition window after therapy. However, although efficient, STP methods offer a limited measurement window because of their reliance on the time dependency of the scan time point.

The iSTP method aims to overcome these limitations arising from the STP, enabling early and flexible posttherapy scan times. It provides advantages for dosimetry assessment instantaneously after the patient starts the treatment, which is not practical because of the logistic challenges (5,8,13–15). iSTP can be achieved based on the knowledge that *T*_eff_ is an intrinsic characteristic of a patient’s health status, integrating both the physical and the biologic half-life processes. The pretherapy patient imaging and clinical data provided valuable information about the patient’s physiologic processes such as metabolism, clearance, and excretion (24), which can be used as features for predicting *T*_eff_. Our investigation also confirms that these predictions, with an ME below 27%, have limited variations even during the therapy cycles, which introduced more physiologic alterations given sufficient pretherapy data, that is, data acquired ideally shortly before the treatment. Additionally, the inclusion of the SUV_mean_ from the urinary tract (kidneys and urinary bladder) and the clinical evaluation of the patient’s organ conditions provided valuable insights for the model, as their classification significantly enhanced the prediction accuracy. The organ’s TIA results obtained from iSTP compared with MTP in relative absolute differences, at different time points (2, 20, 43, and 69 h), for ^177^Lu-PSMA I&T are presented in Figure 3.

Scan time limitation makes STP methods not feasible for dosimetry assessment at 2 h *t*_sc_ (5,12,25). Our iSTP method achieved a significant mean RAD of less than 27% for the left and right kidneys and liver, with differences below 20% for the same organs at 20 h *t*_sc_. From a dosimetry perspective, we demonstrated the potential of this method for early and flexible scan acquisition for dose assessment compared with the standard MTP method.

By applying the Hänscheid method, based on the availability of this retrospective data, our findings align with the optimal performance reported in the literature for both kidneys (8,26). Although 2 h *t*_sc_ is not part of the Hänscheid method, we used it as a reference to demonstrate the instant capability of our method. In Figure 3, we observe that the iSTP method outperformed the conventional method at 2 h, achieving statistical significance (*P* < 0.001) for all organs. Additionally, in Supplemental Figure 1, we show a comparison of the results between the early scan times at 2 h and 20 h for the iSTP method and the Hänscheid method at optimal time points (69 h for the left and right kidneys and 20 h for the liver and spleen). Our analysis revealed that iSTP early time points showed significant differences compared with another method for the left and right kidneys (*P* < 0.05). A similar pattern was seen for the liver at 2 h, though at 20 h, the difference was not significant (*P* = 0.07). No significant difference was observed for the spleen at either time point. Overall, our method performed comparably or better than the Hänscheid method, demonstrating an improved accuracy in certain scenarios considering time point dependency.

Contrary to conventional STP methods, which suffer from large dosimetric variances in the first hours, our method demonstrated an early scan posttherapy point of choice for the left and right kidneys, liver, and spleen. However, significant outliers were identified. Notably, at 2 h *t*_sc_ for both kidneys, outliers were identified in a few patients with differences exceeding 60%. We noted that 1 patient had liver metastasis, normal function in the right kidney, and bone metastasis after treatment. Although these unexpected values may arise from factors such as patient clinical status and estimation of *T*_eff_, suggesting a low level and rapid clearance of activity uptake at earlier time points, they may be associated with fitting and prediction errors. The study by Xue et al. (20) illustrated that the liver exhibited the highest level of radiopharmaceutical accumulation within the body, showcasing the most rapid decrease in radioactivity. The same behavior was observed for the spleen. Such pharmacokinetic inaccuracies potentially compromise the reliability of radiation dose assessments for the liver and spleen at 69 h *t*_sc_ (13,27). Early time points are less influenced by metabolic and clearance processes that can introduce variability. The activity of the radiotracer is higher at early time points, resulting in a better signal-to-noise ratio for imaging and measurements. As the radiotracer decays over time, the signal diminishes, and the influence of background noise and statistical fluctuations increases, leading to significant variability and impracticality of dosimetry at late time points (Supplemental Fig. 3). Highlighting the significance of activity uptake is crucial for understanding the impact it has on the dosimetry process.

The salivary gland would ideally be a key focus as an organ at risk in PSMA RPT. In this retrospective study, it was not included in the field of view during the SPECT acquisition. Nonetheless, the proof of concept in current normal organs paves the way for future studies on other organs using this clinically friendly dosimetry approach.

In the second scenario, the SVR model predicted the *T*_eff_ with an ME ranging from 20% to 32%, averaging a 4% difference from the first scenario for the left and right kidneys and being similar to that for the liver and spleen. However, with the dosimetry analysis grouped by cycle in both scenarios, no statistically significant differences were observed in all grouped cycles. Nonetheless, the mean RAD in absorbed dose indicated a minimum value for the left kidney in cycle 4 and further and a maximum for the liver for the same cycle. This scenario would enable a careful evaluation of using the predicted *T*_eff_ obtained before the first cycle for dosimetry in subsequent cycles. However, to assess the robustness of the model, further validation of our data is required.

The STP method has its limitations in accurately assessing dosimetry at early and later postinjection *t*_sc_, as mentioned previously. On the other hand, the iSTP method targets its application for early time points optimally within the first or even second day after injection, which is generally more practical for dosimetry implementation in outpatient or short-duration inpatient treatment. This is still a challenge for predicting *T*_eff_ for the late cycles. A common assumption that biokinetics and dosimetry remain constant across cycles is a debated topic. Supporting this, Malcom et al. (28) found a 30% increase in kidney *T*_eff_ between cycles, significantly affecting dose predictions.

This study faced several limitations. First, we acknowledge that the small sample size significantly affected the results, leading to inaccurate predictions, fitting issues, and higher variations. We observed more outliers than the conventional STP method. The machine learning model can be improved by incorporating more patient data for training. Future work should aim to increase the sample size to achieve a more robust statistical power. Second, our patient data are limited to 1 bed position of the abdominal region and do not include all critical organs and lesions. Future work that includes other organs and lesions is desired. Despite these limitations, our preliminary findings highlight the potential of the proposed iSTP method to simplify and promote routine clinical dosimetry implementation.

## CONCLUSION

The proposed iSTP method simplifies dosimetry and demonstrates flexibility in selecting early posttreatment imaging time points. Preliminary results indicate that, despite fluctuations in relative absolute differences, our iSTP method effectively predicts the *T*_eff_ using pretherapy data and facilitates STP measurement shortly after RPT. This method could expedite the broader application of dosimetry in both outpatient and short-duration inpatient treatments.

## DISCLOSURE

Financial support was funded by Swiss National Science Foundation (grant no. 188914). Kuangyu Shi and Axel Rominger receive research grants from Novartis and Siemens Healthineers. No other potential conflict of interest relevant to this article was reported.

## Notes

KEY POINTS **QUESTION:** Can early postscan imaging be used for STP dosimetry? **PERTINENT FINDINGS:** Our study demonstrated that incorporating pretherapy data from each cycle and before the first therapy cycle enabled the prediction of organ-specific *T*_eff_ for PSMA RPT. The iSTP method was evaluated against MTP and STP approaches, highlighting its achievement at early and flexible 2 h and 20 h *t*_sc_ for dosimetry after therapy. However, careful evaluation is advised when using PET/CT data from before the first therapy cycle to predict outcomes in subsequent cycles. **IMPLICATIONS FOR PATIENT CARE:** The iSTP method paves the way for improved patient care by ensuring dosimetry assessment is completed before the patient leaves the center after treatment administration.
